# *Citrus sinensis* Peel Oil Extraction and Evaluation as an Antibacterial and Antifungal Agent

**DOI:** 10.3390/microorganisms11071662

**Published:** 2023-06-26

**Authors:** Tauseef Anwar, Huma Qureshi, Arooj Fatima, Kanwal Sattar, Gadah Albasher, Asif Kamal, Asma Ayaz, Wajid Zaman

**Affiliations:** 1 Department of Botany, The Islamia University of Bahawalpur (Baghdad ul Jadeed Campus), Bahawalpur 63100, Pakistan; fatimaarooj712@gmail.com (A.F.); kanwalsattar70@gmail.com (K.S.); 2 Department of Botany, University of Chakwal, Chakwal 48800, Pakistan; huma.qureshi@uoc.edu.pk; 3 Department of Zoology, College of Science, King Saud University, Riyadh 11451, Saudi Arabia; 4 Department of Plant Sciences, Faculty of Biological Sciences, Quaid-i-Azam University, Islamabad 45320, Pakistan; kamal@bs.qau.edu.pk; 5 Faculty of Sports Science, Ningbo University, Ningbo 315211, China; asmaayaz@bs.qau.edu.pk; 6 Department of Life Sciences, Yeungnam University, Gyeongsan 38541, Republic of Korea

**Keywords:** *Citrus sinensis*, antimicrobial effectiveness, minimum inhibitory concentration, natural preservative, industries

## Abstract

Throughout the tropical and subtropical climates, the genus Citrus can be found. The current study was conducted to extract the *Citrus sinensis* peel oil and evaluate its antibacterial, antifungal and antiparasitic potential. Petroleum ether was used to extract the *C. sinensis* peel oil through a Soxhlet apparatus. The antimicrobial and antifungal potential was determined via agar well diffusion method and minimum inhibitory concentrations (MIC) were calculated (test bacterial strains: *Staphylococcus aureus*, *Escherichia coli* and *Streptococcus agalactiae*; test fungal strains: *Aspergillus flavus*, *Aspergillus niger*, *Altrnaria alternata*). Antiparasitic activity against *Leishmania* *tropica* was determined following standard protocol using amphotericin-B as positive and Dimethyl Sulfoxide (DMSO) as a negative control and the percentage inhibition was calculated. The oil extracted was brownish yellow with a tangy smell, water-insoluble, density (0.778 g/cm^3^) and specific gravity (0.843 g/cm). In antibacterial activity, the diameter of the zone of inhibition was maximum against *E. coli* (14 mm) and minimum for *S. agalactiae* (10 mm). While in antifungal activity diameter of the zone of inhibition was maximum against *A. flavus* (12.5 mm) and minimum for *A. alternata* (8.6 mm). *S. agalactiae* exhibited the minimum MIC value (6 mg/mL) and in fungal strains *A. alternata* exhibited the minimum value (2 mm). *Citrus sinensis* peel oil displayed antileishmanial efficiency of 60% at 50 μg/mL concentration after 48 h of incubation. *C. sinensis* peel oil demonstrated antimicrobial capabilities, implying that it could be used as a natural preservative in food or as an effective treatment against a variety of pathogenic organisms. Industries should extract oil from the waste of citrus fruits which will be beneficial from an economic point of view.

## 1. Introduction

In many developing countries today, microorganisms are regarded as the main cause of diseases and mortality. Despite advances in diverse antimicrobials made by pharmaceutical endeavors, the protection from antitoxins has increased overall in many bacterial microorganisms [1]. Antibacterial resistance is rapidly rising. Due to the development of a strong defense mechanism against antibiotics, the incidence of antibiotic resistance is a persistent issue. As a result, it’s essential to utilize and create innovative inhibitory compounds to combat resistant microbial infections [2]. There is a dire and persistent requirement for finding and examining novel antimicrobial compounds. Centers for Disease Control and Prevention (CDC) reports that two million individuals are impacted by complicated issues from antibiotic-resistant pathogens. Due to this pathogenicity, about 23,000 people pass away yearly in the United States [3]. The following restriction applies to synthetic antibiotics: First, these are expensive and unaffordable for patients from developing nations. Second, as time goes on, bacteria develop resistance to antibiotics. Consequently, many antibiotics lose their effectiveness against microorganisms over time. On the other hand, natural substances have been remarkably effective in serving as a standard for the creation of brand-new antibacterial medications. Additionally, these methods of producing antibiotics are biocompatible [4].

As is commonly acknowledged, bioactive plant extracts represent a potential source for the majority of medications. For instance, the plant-derived antibiotics quinine (Cinchona) and berberine (Berberis) are quite effective against microorganisms (*Staphylococcus aureus* and *Escherichia coli*) [5]. In order to find plant-based medications, a range of strategies have been established, including biological screening, isolation, and clinical trials for various plants. There are numerous articles that demonstrate the herbs employed in conventional therapies are viable against microorganisms. Plant extracts have a negative impact on the cellular structures of microorganisms. It is carried out in a variety of ways, starting with a pharmacological attack on the target cell’s cell membrane. The bilayer of proteins and lipids that makes up a cell membrane is impacted as a result of their action. Since antiquity, many plant extracts have been used, and today more research is being done on their potential to improve health, have therapeutic benefits, and possibly even serve as a means of preventing certain diseases. Essential oils are frequently employed in human and animal therapy in addition to plant extracts. They have been used and renowned for their antibacterial qualities for millennia [6].

According to the most recent research, essential oils are one of the substances that are being used more and more frequently to manage pests, such as bacteria, insects, and fungi-plant diseases [7]. Methods including hydro-distillation (HD), solvent extraction (SE), and steam distillation (SD), are used to obtain these oils [8]. The citrus plant belongs to the Rutaceae family, which also includes about seventeen different species and is spread throughout the following three climate zones: tropical, subtropical, and temperate. Around 140 countries annually produce 70 million tons of citrus. Pakistan is the 12th-largest producer of citrus, with a yearly production of over 1,816,000 tons [9]. Peels and bagasse, which make up 40–50% of the total fruit mass, are produced in large quantities during the production of citrus juice [10]. However, improper removal of citrus peels will not only result in resource abuse but also in climatic damage, highlighting the importance of careful utilization of citrus wastes. Citrus peel essential oils provide a variety of health benefits, including the ability to reduce hypercholesterolemia and act as a diuretic, and antihypertensive. As a waste management strategy, essential oil from citrus peel can be extracted. The current study was designed to demonstrate the yield of citrus peel oil using a simplified method of extraction through the Soxhlet apparatus [11] and assess its antibacterial, antifungal and antiparasitic effectiveness.

## 2. Materials and Methods

### 2.1. Study Area

In the biochemistry laboratory of the Department of Physiology and Biochemistry at Cholistan University of Veterinary and Animal Sciences (CUVAS), Bahawalpur, Pakistan, essential oil derived from *C. sinensis* peels was extracted and tested for antibacterial, antifungal and antiparasitic activity from November 2021 to May 2022.

### 2.2. Collection and Extraction of Oil

**Figure 1:**
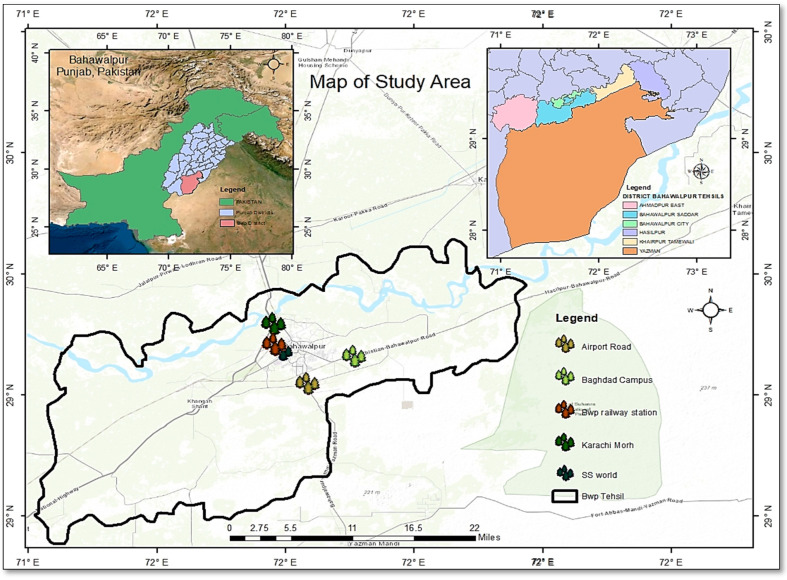
Collection sites of fresh citrus peels.

Fresh citrus peels were collected from the local juice corners of five different locations in Bahawalpur i.e., Airport Road (29.3509° N, 71.7109° E), Islamia University, Baghdad ul Jadeed campus (71.7593° N, 71.71120° E), Karachi Morh (29.3833° N, 71.0333° E), Railway station (29.395721° N, 71.683334° E) and SS world park (29.3937° N, 71.6618° E) (Figure 1).

**Figure 2:**
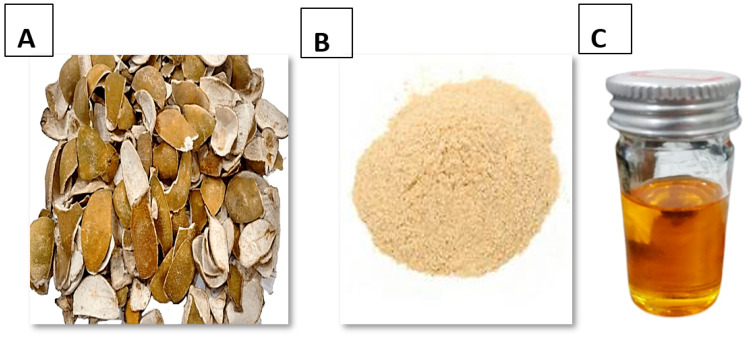
*Citrus sinensis* peels (**A**) Dried, (**B**) Powder, (**C**) Oil.

After collection, the peels were cleaned and washed with distilled water. The peels were then dried in the shade until removal of all moisture content and powdered with the help of pestle and mortar instead of using an electric grinder which cause the loss of useful components due to rotation at high speed (Figure 2A,B). The oil was extracted by using the solvent extraction method (Figure 2C). For this purpose, the Soxhlet apparatus was used with solvent petroleum ether. The 200 mL of petroleum ether was measured and poured into a flask. The 10 g powder of citrus peel was weighed and dropped into an extractor by the thimble. The yield of oil was noted at different temperatures. A separatory funnel was used to separate the oil from the solvent.

Pure citrus oil was obtained after this process. Oil yield was calculated by the given equation:

(1) $$Yield\left(\%\right)=\frac{Weight\;of\;extracted\;oil}{Weight\;of\;sample\;used}\times 100$$

### 2.3. Evaluation of Physical Properties

The physical properties of color, odor, density, solubility and specific gravity of *C. sinensis* oil were determined using equations:

(2) $$\mathrm{Density}\;\mathrm{of}\;\mathrm{oil}=\frac{Weight\;of\;sample}{Volume\;of\;sample}\times 100$$

(3) $$\mathrm{Specific}\;\mathrm{gravity}\;\mathrm{of}\;\mathrm{oil}=\frac{Weight\;of\;oil\;extract}{weight\;of\;water}\times 100$$

### 2.4. Qualitative Analysis of Citrus Oil

The components of orange essential oil were separated using a capillary column (Rtx-1.30 m 0.32 mm I.D., 0.25 µm) and a GC-MS (Shimadzu-QP-2010S plus (Kyoto, Japan)) instrument in a sample solution of 1 µL (100 µg extract/mL) in a 1:1 hexane: diethyl ether ratio. The oven temperature was set for a starting temperature of 50 °C, a 5 °C/min temperature ramp to 180 °C, which was held for 1 min, then a 10 °C/min temperature increase to 250 °C. The column flow of the helium carrier gas (He) was 2.62 mL/min with a linear velocity of 58.7 cm/s. The following mass parameters were used to acquire the compounds: scan mode ACQ start of *m*/*z* 70 and finish of *m*/*z* 500; solvent cut time of 4.0 min; start time of 4.1 min; and end time of 34.4 min. NIST LIBRARY 2020 (Shimadzu, Tokyo, Japan) and Lab Solution software 4.1 for GC solution Ver. 2.5 were used to do the integration and comparison of the compounds.

### 2.5. Antibacterial Assay

#### 2.5.1. Microbial Organisms

**Figure 3:**
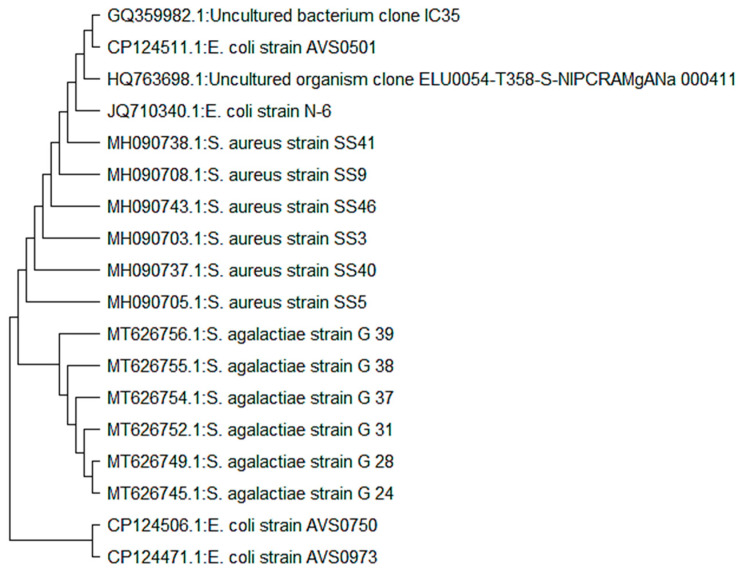
Phylogenetic trees of bacterial isolates (*E. coli*, *S. aureus* and *S. agalactiae*).

The essential oil was tested against three bacterial strains: *Staphylococcus aureus* (MH090705), *Escherichia coli* (JQ710340) and *Streptococcus agalactiae* (GQ994974). Bacterial cultures were maintained on agar nutrient media. The phylogenetic tree of test species was constructed using MegaX software (Figure 3).

#### 2.5.2. Agar Well Diffusion Method

The standard well diffusion method recommended by the Clinical & Laboratory Standards Institute (CLSI) was used to determine the antibacterial properties of *C. sinensis* oil. Bacterial species were first inoculated into nutrient agar medium and incubated overnight at 37 °C and checked for purity. For the preparation of media, 14 g nutrient agar was dissolved in 500 mL of distilled water and autoclaved at 121 °C for 15 min. 4 mL of medium was poured into three sterilized petri plates and closed immediately. Sodium chloride solution (0.9%) was used to create all bacterial suspensions at a standard density of 0.5 McFarland (1108 cells per mL; Bio-Mérieux, Marcy I’Etoile, France). Prepared bacterial suspensions were spread on the agar media plates by sterile swab. Wells were made on agar plates with the help of inoculating loops. Three hundred and sixty microliters of the citrus oil were impregnated into 640 microliters DMSO and then it was vortexed. The diluted solution was added to wells with the help of a pipette. 30 microliters were added to each well. For 24 h, all the examined microorganisms were cultured at 37 °C. The inhibitory zones’ sizes were measured in millimeters. The assays were carried out three times each.

#### 2.5.3. Minimum Inhibitory Concentration

The minimum concentration of drug which inhibits microbial growth which is expressed in mg/µL is called MIC. The activity was performed on 96-well microliter plates. The minimal inhibitory concentration was calculated using the broth dilution technique. For the preparation of broth media, 14 g nutrient broth was added in 500 mL distilled water and autoclaved at 121 °C for 15 min. Hundred µL broth was added to each well of 96-well titration plate. Fifty µL of bacterial solutions were added in wells 2–12 in the plate. Then 50 µL of extracted oil was added to the first column wells of the plate. After that two-fold dilution was made. As a result, the first well contains only broth media while the last well contains a mixture of broth and bacteria. All the other wells contain a mixture of broth, citrus oil and bacteria. The serial dilution was also done before the addition of bacteria. Plates were incubated for 24 h at 37 °C. The first reading was taken and the second one was taken after 24 h. Readings were taken using the Elsevier reader.

### 2.6. Antifungal Assay

#### 2.6.1. Microorganisms and Nutrient Media

**Figure 4:**
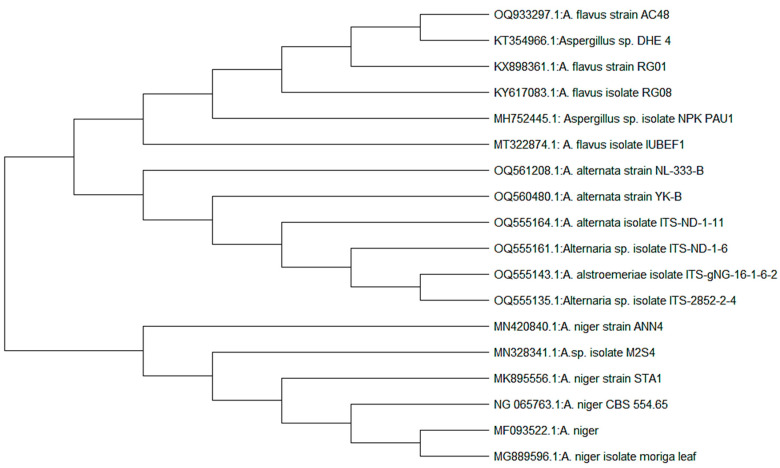
Phylogenetic trees of fungal isolates (*A. niger*, *A. flavus* and *A. alternata*).

The essential oil was tested against three fungal strains: *Aspergillus niger* (MK895556), *Aspergillus flavus* (MT322874), and *Alternaria alternata* (MK834822). Fungal cultures were maintained on a potato dextrose agar nutrient medium. A phylogenetic tree of fungal strains was constructed using MegaX software (Figure 4).

#### 2.6.2. Agar Well Diffusion Method

The agar well diffusion method was used as a preliminary assay for testing the antifungal effect of citrus oil. Potato dextrose agar (PDA) media sterilized in a flask and cooled to 45–50 °C was poured into sterilized petri dishes with a diameter of 8 cm. A suspension of the tested fungal spores (2 × 10^5^ spores) was spread on media via cotton swab. The wells are made on an agar plate with the help of inoculating loops. Citrus oil was diluted by adding 360 µL of citrus oil in 640 µL DMSO and vortexed. This diluted solution was added to wells with the help of a pipette. A 30 µL solution was added to one well. The plates were incubated at 37 °C for 24 h for maximum growth of microorganisms.

#### 2.6.3. Minimum Inhibitory Concentration (MIC)

The broth dilution method was used to determine the minimum inhibitory concentration. For the preparation of broth media, 14 g nutrient broth was added in 500 mL distilled water and autoclaved at 121 °C for 15 min. Hundred µL broth was added in each well of 96-well titration plate with the help of a pipette. Fifty µL of fungal solution was added in wells 2–12 in the plate. The 50 µL of oil was added to the wells’ first column of plate. After that two-fold dilution was made. As a result, the first well contains only broth media while the last well contains a mixture of broth and fungi. All the other wells contain a mixture of broth, citrus oil and fungi. The plates were incubated at 37 °C for 24 h. The first reading was taken at that time while the other was after 24 h. The Elsevier reader was used to take readings.

### 2.7. Anti-Parasitic Potential

The antiparasitic potential was assessed against *Leishmania* *tropica* promastigotes, following standard protocol with a little modification [12]. All testing tubes had 3 mL of medium with 1 × 10^5^ parasites/mL of *L. tropica* promastigotes. In each tube, 5 mL of each concentration (6.25 μg/mL, 12.5 μg/mL, 25 μg/mL, and 50 μg/mL) of citrus oil were placed in an incubator at 25 °C in the oven. In the whole trial, amphotericin-B was used as a positive and DMSO was used as a negative control. Parasites were counted by hemocytometer in both control and oil-treated tubes at various intermissions 12, 24, 48 and 72 h of incubation and the percentage inhibition was confirmed applying the following formula:

(%) Inhibition = 100 × Ab sample/Ab control(4)

In the equation,

Ab sample = absorbance of the tested sample (citrus oil)

Ab sample = absorbance of the negative control.

## 3. Results and Discussion

### 3.1. Physical Properties of Essential Oil

The findings of physical characterization are shown in Table 1. The oil extracted by the Soxhlet extraction method was brownish-yellow in color with a tangy smell. Specific gravity is the ratio of a material’s density to that of water. The specific gravity of citrus oil was 0.843 g/cm^3^. The density of oil was 0.778 g/cm^3^. The oil was insoluble in water and floated over due to its lesser density than water. The physical and chemical characteristics of citrus peel oil have been studied previously [13]. The density of oil was 0.821 which in the present study was recorded as 0.778 g/cm^3^. The color and specific gravity observed were also quite similar as the color was orange-yellow and the gravity was 0.82 g/cm^3^. In this study, the major constituent of citrus peel includes cellulose, hemicellulose, lignin, pectin (galacturonic acid) and other low molecular weight compounds viz; β-pinene (0.55%), limonene (96–98%), α-pinene (0.29%), myrcene (1.3–1.45%) and octanol (0.37–0.53%).

In another study, the seed and peel oil of *C. sinensis* were compared for their physical properties. The results showed that the seed oil of *C. sinensis* had golden yellowish color while the peel oil showed brownish yellow color. This is fairly in agreement with our study where the peel oil exhibited brownish-yellow color similar to the yellow color obtained in grapefruit oil [14]. The relative density of the oil was 0.778 g/cm^3^. Although it is lesser than the value reported by other researchers. The essential oil had a specific gravity value < 1 except a few oxygenated aromatic compounds. The extracted oil had a specific gravity of less than 1 showing that the oil is lighter than water and is insoluble in water [15]. This fact agreed with current findings where the essential oil had a specific gravity value of 0.843 g/cm^3^ which was less than 1.

### 3.2. Effect of Temperature on Percentage Yield

The effect of temperature during the extraction process on the percentage yield of oil was also calculated. The volume of oil extracted from the citrus peels at different temperatures was different. Table 2 shows the extraction of oil at temperatures of 50–80 °C with the solvent petroleum ether. The oil yield was calculated as 1.2 mL, 1.6 mL, and 1.8 mL at 50 °C, 60 °C and 80 °C temperatures, respectively. The highest value of oil extracted was observed at 80 °C. The percentage yield of oil at 50, 60 and 80 °C was, respectively, 2.4%, 3.2% and 3.6%. An increase in extraction temperature leads to a corresponding increase in essential oil yield [16]. The maximum temperature in the study was 90 °C at which the oil yield was recorded maximum. This is fairly in agreement with the present study as it reported that the amount of oil extracted was different at different temperatures. Previous research also confirmed that an increase in temperature up to the optimum level causes an increase in the yield of extracted oil [17].

**Figure 5:**
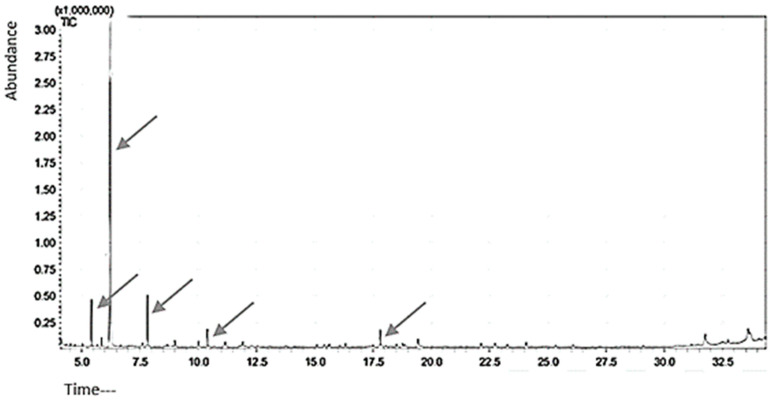
Orange peel powders’ essential oil constituents as determined by GC-MS (compound **1**: β-Myrcene, compound **2**: D-Limonene, compound **3**: β-Linalool, compound **4**: Decanal, compound **5**: Valencene). (Arrows used for peaks).

Five peaks were visible on the GC-MS chromatogram of the citrus peel oil extract, indicating the presence of five different compounds. Figure 5 lists the compounds discovered. D-Limonene (95.7%) was shown to be the main component by GC-MS analysis. More than 70% limonene is often present in citrus species peels. A total of 65% of limonene was found in the current investigation. D-limonene, the oil’s primary ingredient, is likely what gives it its antibacterial and antifungal properties.

### 3.3. Antibacterial Activity of Essential Oil

The in vitro antibacterial activity of *C. sinensis* essential oil against *S. aureus*, *E. coli* and *S. agalactia* was assessed by measuring the zone of inhibition. The data obtained from the well diffusion method indicated that essential oil displayed a significant antibacterial activity on different tested bacterial strains (Table 3). The highest antibacterial activity of this essential oil was observed against *E. coli* with a zone of inhibition of 14.33 ± 2.08 mm. The zone of inhibition for *S. agalactiae* and *S. aureus* were 10.67 ± 1.53 mm and 11.33 ± 1.16 mm, respectively. The application of citrus oil significantly (*p* < 0.05) controlled the growth of all bacterial strains. The order of bacterial strains according to the value of zone of inhibition was *E. coli* > *S. aureus* > *S. agalactiae*. So, *E. coli* has the highest value of zone of inhibition and least growth while *S. agalactiae* has a minimum value of zone of inhibition and the highest rate of growth.

Previous studies revealed that citrus oil contains biological activities including antioxidant, anti-cancer, as well as antimicrobial activities [18]. The antibacterial activity results of citrus oil extracted with petroleum ether agree with previous findings [19]. This may be because these substances have the capacity to harm bacterial cell membranes, making them permeable, and leading to eventual death via ion and molecule leakage. Earlier studies on the antibacterial activity of *Citrus gigantea* leaf extract showed a significant effect on tested organisms. The extract showed a maximum zone of inhibition against *E. coli*. The essential oil from two cultivars of citrus, including *C. hystrix* and *C. aurantifolia* exhibited antibacterial activity against *E. coli* and *S. aureus*. It was evaluated that the petroleum ether extract showed a significant zone of inhibition (10–14 mm) against *E. coli*, *S. aureus* and *S. agalactiae*. Results are supported by the study where it was reported that the petroleum ether extract of some plant leaves can be effective against tested bacteria *E. coli* and *S. aureus* [20]. The antibacterial activity of peel extracts of *C. sinensis* oil with petroleum ether was evaluated against different bacteria. It showed that the oil was more effective against gram-negative bacteria than gram-positive which agreed with previous studies but contrary to many other researches where it was more effective against gram-positive than gram-negative due to the fact that the walls of gram-negative bacteria are more complex due to lipopolysaccharides layer as compared to gram-positive bacteria [21].

### 3.4. Determination of Minimum Inhibitory Concentration (MIC) against Bacterial Strains

The results of MIC are shown in Table 4. The data obtained shows that the essential oil exhibited varying levels of antibacterial activity against the investigated bacterial strains. Minimum MIC values were observed in the case of *S. agalactiae,* i.e., 6.51 mg/mL. While MIC values for *Staphylococcus aureus*, *Escherichia coli* were 10.41 ± 4.51 and 13.02 ± 0.00 mg/mL, respectively. In the present study, the citrus oil was more effective against the gram-positive bacteria at 6 mg/mL for *S. agalactiae* and 10 mg/mL against *S. aureus* as compared to gram-negative bacteria at 13 mg/mL against *E. coli* this was due to the fact that the walls of gram-negative bacteria have high phospholipid content and lipopolysaccharides which is more complicated as compared to gram-positive bacteria. Lower MIC value means higher efficiency of the extract against the bacteria. The minimum concentration of citrus oil at which the growth of bacteria was suppressed was against *S. agalactiae* rather than *E. coli* and *S. aureus.* The order of bacterial strains according to MIC value was *S. agalactiae* > *S. aureus* > *E. coli*. So, *S. agalactiae* had the lowest MIC value of citrus oil that did not allow the visible growth of bacteria. The results indicated that citrus peel oil with maximum MIC value was not much effective against *E. coli*. The lowest concentration of the extract inhibits the growth of bacteria (no observable growth of bacteria) and is expressed in mg/mL [22]. The minimum inhibitory concentration of essential oil extracted from *Citrus hystrix* (kaffir lime) peels was evaluated against the six bacterial pathogens via the broth microdilution method. The results exposed that gram-positive bacteria showed the maximum efficiency to hinder or kill the visible growth of bacteria [23].

### 3.5. Antifungal Activity

The in vitro antimicrobial activity of citrus oil against *Aspergillus flavus*, *Aspergillus niger* and *Altrnaria alternata* was assessed by comparison of inhibition zone diameters. The data obtained from the well diffusion method (Table 5) indicated that essential oil displayed a significant antifungal activity. The highest antifungal activity of essential oil was observed against *A. flavus*, with a zone of inhibition of 12.5 mm. The zone of inhibition for *A. alternata* and *A. niger* were 8.6 mm and 7.00 mm, respectively. The application of citrus oil significantly (*p* < 0.05) controlled the growth of all fungal strains. The sequence of their action was *A. niger* < *A. alternata* < *A. flavus*. Orange peel oil was found to be the most effective against *Aspergillus niger* and *Penicillium* spp. At all three concentrations (30, 60, and 90%), while lime was effective at higher concentrations of 60 and 90%, and lemon peel essential oil was completely resisted by *S. aureus* [24]. In comparison to the other species, lime peel oil inhibited *Aspergillus niger* the most. *Citrus limon* essential oil has potential against *A. flavus*, *A. fusarium* and *A. niger* with a zone of inhibition 11 mm, 8 mm and 7 mm, respectively. The application of citrus oil reduces fungal growth significantly. Previously the application of different oils has been reported. The research showed a high diversification of the influence of *C. sinensis* EO on microorganisms. It was shown that the oil was most effective in limiting the growth and sporulation of microbes [25]. The antimicrobial activity of essential oil is probably due to the presence of a single compound, synergism, or antagonism among several compounds.

### 3.6. Determination of Minimum Inhibitory Concentration against Fungal Strains

The results of MIC are shown in Table 6. The minimum MIC value was showed by *A. alternata* which was 2.50 ± 0.00 mg/mL, *A. niger* MIC value was at second and noted as 6.67 ± 2.89 mg/mL. The highest MIC values were shown by *A. flavus* 8.33 ± 2.89 mg/mL. Its CI values were 3.34 and 10.00. In a study *C. sinensis*, *C. limon*, *C. paradise* oils were tested for antimicrobial activity in which *A. alternata*, *A. niger*, *A. flavus* were observed to be affected more [26]. For, *A. alternata* MIC value was recorded as 3.01 while in the current study, its value is 2.50 ± 0.00 mg/mL. This indicates that at this minimum value, the oil obstructs growth in both cases. For *A. niger* and *A. flavus* the values were observed higher than the other five species [27]. The full factorial design was used to measure the concentrations of caracole, thyme oil and *C. reticulata* oil. The three extracts showed different activity against selected fungal strains. The results in the case of *C. reticulata* were quite similar to current findings.

### 3.7. Antileishmanial Potential

**Figure 6:**
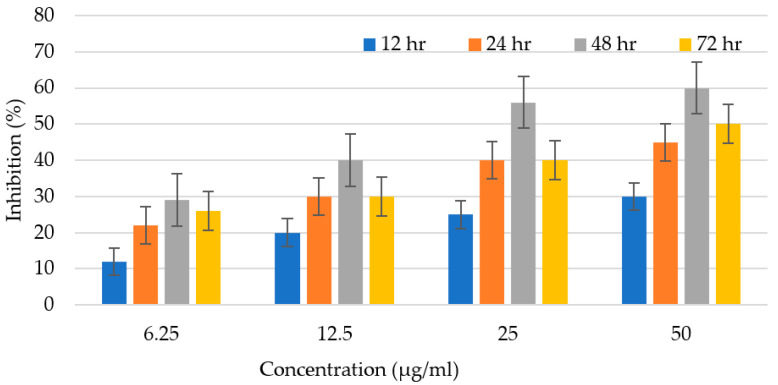
Antileishmanial activity of citrus oil at various concentration at different time intervals.

The efficiency of *C. sinensis* peel oil as an antileishmanial agent was observed for 72 h and mentioned as % inhibition (Figure 6). Promastigotes number were counted in both experimental and control groups at various time intervals (12, 24, 48, and 72 h). After amendment with multiple doses of the citrus oil, the parasite number was counted in treated and control trials at consistent time duration extending from 12–72 h. The anti-leishmanial potential is enhanced with the rising concentration of oil. The antileishmanial potential was examined to be 14% at 6.25 μg/mL, 20% at 12.5 μg/mL, 25% at 25 μg/mL, and 30% at 50 μg/mL, in the initial incubation for 12 h. *C. sinensis* peel oil displayed antileishmanial efficiency of 30%, 40%, 55%, and 60% at 6.25 μg/mL, 12 μg/mL, 25 μg/mL, and 50 μg/mL, respectively, after 48 h of incubation. After that, there was a slight decrease in activity. Afterward, a little decrease in potential was observed. Citrus oil has the ability of ROS production, which destroys pathogens by creating holes in the cell wall and affects the physical properties of the cell membrane, causing the bursting of the membrane and leakage of intracellular constituents. It is also documented that *Leishmania* is extremely sensitive to these ROS, and the medicine that would produce ROS will be acknowledged as an effective antileishmanial agent. Another important factor that can lead to a good performance of antiparasitic activity is either the synergism of the major chemical constituents found in the essential oil or the presence of other constituents that can also be active at even lower concentrations [28]. Previously scientists have described that α-pinene has recognized leishmanicidal activity against *L. brasiliensis* though this terpene is at a concentration below 10% [29]. Furthermore, the reports have shown that β-caryophyllene (14.0%) is also vigorous against *Leishmania* genus [30]. The substantial findings of our research thus absolutely show that citrus oil may be a capable agent for leishmaniasis treatment.

Based on these results, analysis of nanoformulations of the same treatments with citrus oil presented in this paper is recommended as was carried out with *Brucea javanica* oil [31]. Zinc oxide biosynthesized nanoparticles using *Ziziphus jujuba* leaves extract showed effective antibacterial activities against *S. aureus* and *E. coli* bacteria, as the inhibition zones were 15 mm and 11 mm for the *E. coli* and *S. aureus*, respectively, and had an outstanding cytotoxic effect on cancer cell lines [32]. Chemical characterization and analysis of individual compounds is further suggested as done with *Pulicaria jaubertii* where six isolated flavonoids exhibited antiproliferative activity against the HCT-116 cell line with IC_50_ values at 33 ± 1.25, 36 ± 2.25, 34 ± 2.15, 32 ± 2.35, 34 ± 2.65, and 36 ± 1.95 μg/mL for compounds 1 to 6, respectively [33].

## 4. Conclusions

The oil extracted from *C. sinensis* fruit peels through the Soxhlet apparatus was brownish-yellow in color with a tangy smell. The antibacterial potential of oil against bacterial strains i.e., *E. coli*, *S. aureus* and *S. agalactiae* exhibited that the oil efficiently controls the bacterial growth. The highest zone of inhibition was observed against the strain *E. coli*. The antifungal potential of oil for pathogenic fungal strains i.e., *A. flavus*, *A. niger*, *A. alternata* showed that the oil significantly inhibits fungal growth. The diameter of the zone of inhibition was maximum against *A. flavus*. Notably, phytoactive chemicals found in citrus oil may serve as prototypes in the production of novel anti-microbial and anti-parasitic medications. Still, plants are regarded as auspicious sources in the field of medicinal advancements. Citrus fruit waste should be processed into oil by industries since doing so will be profitable. Based on current results, chemical characterization and nanoformulations analysis are recommended.

## Figures and Tables

**Table 1:** Physical properties of *Citrus sinensis* peel oil.

Parameters	Results
Odor	Tangy smell
Color	Brownish yellow
Density	0.778 g/cm^3^
Solubility	Insoluble in H_2_O
Specific gravity	0.843 g/cm^3^

**Table 2:** Effect of temperature on percentage yield of *Citrus sinensis* peel oil.

Solvent	Weight of Peels (g)	Volume of Solvent (mL)	Temperature	Time (min)	Quantity of Oil Extracted (mL)	Percentage Yield of Oil
Petroleum Ether	50	300	50	240	1.2	2.4
	50	300	60	240	1.6	3.2
	50	300	80	240	1.8	3.6

**Table 3:** Antibacterial activity of *Citrus sinensis* peel oil against bacterial strains.

Bacterial Strains	Zone of Inhibition (mm)	Minimum	Maximum
*Escherichia coli*	14.33 ± 2.08 *^a^	12	16
*Staphylococcus aureus*	11.33 ± 1.16 *^b^	10	12
*Streptococcus agalactiae*	10.67 ± 1.53 *^b^	9	12

* Different superscripts in column showed significant-difference in comparison to blank (*p* < 0.05).

**Table 4:** Minimum Inhibitory concentration (mg/mL) of *Citrus sinensis* peel oil against bacterial strains.

Bacterial Strains	MIC (mg/mL)	Minimum	Maximum
*Escherichia coli*	13.02 ± 0.00 *^a^	7.810	15.620
*Staphylococcus aureus*	10.41 ± 4.51 *^b^	7.810	15.620
*Streptococcus agalactiae*	6.51 ± 2.26 *^c^	3.900	7.810

* Different superscripts in column showed significant-difference (*p* < 0.05) in comparison to control.

**Table 5:** Antifungal activity of *C. sinensis* oil against fungal strains.

Fungal Strains	Zone of Inhibition	95% CI
*Alterneria alternata*	8.667 ± 1.528 *^b^	(6.828, 10.506)
*Aspergillus flavus*	12.500 ± 1.323 *^a^	(10.661, 14.339)
*Aspergillus niger*	7.000 ± 1.00 *^b^	(5.161, 8.839)

* Different superscripts within column showed significant-difference (*p* < 0.05).

**Table 6:** Minimum Inhibitory concentration (mg/mL) of *Citrus sinensis* peel oil against fungal strains.

Fungus	MIC (mg/mL)	95% CI
*Alterneria alternata*	2.500 ± 0.000 ^a^	(−0.830, 5.830)
*Aspergillus flavus*	8.33 ± 2.89 ^a^	(5.00, 11.66)
*Aspergillus niger*	6.67 ± 2.89 ^a^	(3.34, 10.00)

Different superscripts within column showed significant-difference (*p* < 0.05).
